# Maternal salinity influences anatomical parameters, pectin content, biochemical and genetic modifications of two *Salicornia europaea* populations under salt stress

**DOI:** 10.1038/s41598-022-06385-3

**Published:** 2022-02-22

**Authors:** S. Cárdenas-Pérez, K. Niedojadło, A. Mierek‐Adamska, G. B. Dąbrowska, A. Piernik

**Affiliations:** 1 Chair of Geobotany and Landscape Planning, Faculty of Biological and Veterinary Sciences, Nicolaus Copernicus University, Lwowska 1, 87-100 Toruń, Poland; 2 Department of Cellular and Molecular Biology, Faculty of Biological and Veterinary Sciences, Nicolaus Copernicus University, Lwowska 1, 87-100 Toruń, Poland; 3 Department of Genetics, Faculty of Biological and Veterinary Sciences, Nicolaus Copernicus University, Lwowska 1, 87-100 Toruń, Poland; 4 Centre for Modern Interdisciplinary Technologies, Nicolaus Copernicus University, Wileńska 4, 87-100 Toruń, Poland

**Keywords:** Ecology, Plant sciences, Ecology, Environmental sciences

## Abstract

*Salicornia europaea* is among the most salt-tolerant of plants, and is widely distributed in non-tropical regions. Here, we investigated whether maternal habitats can influence different responses in physiology and anatomy depending on environmental conditions. We studied the influence of maternal habitat on *S. europaea* cell anatomy, pectin content, biochemical and enzymatic modifications under six different salinity treatments of a natural-high-saline habitat (~ 1000 mM) (Ciechocinek [Cie]) and an anthropogenic-lower-saline habitat (~ 550 mM) (Inowrocław [Inw]). The Inw population showed the highest cell area and roundness of stem water storing cells at high salinity and had the maximum proline, carotenoid, protein, catalase activity within salt treatments, and a maximum high and low methyl esterified homogalacturonan content. The Cie population had the highest hydrogen peroxide and peroxidase activity along with the salinity gradient. Gene expression analysis of *SeSOS1* and *SeNHX1* evidenced the differences between the studied populations and suggested the important role of Na^+^ sequestration into the vacuoles. Our results suggest that the higher salt tolerance of Inw may be derived from a less stressed maternal salinity that provides a better adaptive plasticity of *S. europaea*. Thus, the influence of the maternal environment may provide physiological and anatomical modifications of local populations.

## Introduction

Salt stress is one of the main environmental factors that limits growth of plants worldwide. An environment with high, medium or low salinity may impact plants’ ability to tolerate high salinity, and this impact varies between and within species. In heterogeneous environments such as natural and anthropogenic sites, plants could develop multiple strategies through producing offspring that differ in their salt stress tolerance^1,2^. For instance, according to El-Keblawy et al.^3^, the halophyte *Anabasis setifera* with phenotypic plasticity is more able to survive in harsh environments conditions due to the maternal environment being able to produce progeny that fit specific habitats well. Maternal effects might influence adaptive plasticity between generations, which can be considered an adaptive evolution due to the advantage conferred to the offspring reflected by an increased survival^4, 5^. Many studies have demonstrated that maternal habitats can cause plants’ growth to respond differently depending on environmental conditions and, subsequently, this affects the next generation^3, 5, 6^. Some studies have reported contradictory results: for instance, El-Keblawy et al.^3^ showed that the halophyte *Anabasis setifera* has greater salt tolerance when taken from non-saline habitat as compared to a population from a low saline habitat (17.5 mS cm^−1^), while Van Zandt and Mopper^4^ reported that *Iris hexagona* seeds from maternal high salinity germinated earlier and in greater quantity than did seeds from low salinity plants. Currently, many projects for exploiting saline lands are interested in how to predict which genotypes have greater salt tolerance. Therefore, understanding the influence of maternal salinity in the salt tolerance of plants is of great interest.

The main compound that affects a plant’s growth during salinity tolerance is sodium chloride, which is present in many saline soils. High salinity may lower the availability of water to plants in the first phase of development, which relates to the closure of stoma and cell expansion inhibition in shoots^7,8^. This stage is due to an increase in the osmotic force that holds water in the soil, reducing the available water potential. As a result, the reduced availability of water in the soil causes a decrease in cell turgor pressure and a subsequent inhibition of cell elongation in plants such as *Populus tremula*^2^. The influence of salinity on physiology and gene expression has already been reported, but the influence of maternal salinity on cell architecture as a link between biochemical changes and gene expression is still not entirely understood. The cell wall is a dynamic barrier that can vary under the effect of different stimuli that disturb cells, tissues and organisms. High salinity can produce both quantitative and qualitative modifications in the cell wall components. The primary cell wall is composed of three different carbohydrate-based biopolymers, namely hemicellulose, cellulose and pectin. It has been shown that salt stress causes a remodelling in hemicellulose, cellulose^9,10^, pectins^11^ and cell wall proteins^12^. In the primary cell wall of dicots, a major polysaccharide fraction is pectin (∼35%) with a network of cellulose micro fibrils and hemicellulose^13^. Homogalacturonan (HG) is the most abundant pectic polysaccharide in cell wall, constituting 65% of total pectin, followed by xylogalacturonan, apiogalacturonan and rhamnogalacturonan I and II. HG consists of a linear α-1,4-linked galacturonic acid homopolymer and can be methylesterified or de-methylesterified at the C-6 carboxyl group^14^. The de-methylesterified pectate can form Ca^2+^-pectate cross-linked “eggbox” complexes which together with rhamnogalacturonan II affect the cellulose network, which is reflected in a less extensible cell wall that can translate to lower water cell extensibility and can be considered a characteristic of salt-sensitive cultivars^15^. Le Gall et al.^11^ reported that in soybean a severe decrease (~ 75%) in pectin content was observed in the salt-sensitive cultivar, compared to only a 30% decrease observed in the salt-tolerant one.

Additionally, the maintenance of cell turgor, cell’s area and roundness are important parameters describing the physical properties of the cell wall during salt stress. In this sense, proline, as an osmotic regulator, enzyme denaturation protector and macromolecule assembly stabiliser, allows additional water from the environment to be reserved in cells, thereby allowing water potentials to decrease; this can then be observed in an increase in cell area, a large cell area and roundness in plant’s tissue can be associated to the succulence of the plant. As reported in other studies succulence is the ability of some plants to store water and to be somehow independent of external water supply, this can be also linked to the anatomical adaptation during salinity tolerance^16, 17^. Moreover, stomatal conductance is reduced when plants are exposed to high salinity, which leads to the generation of reactive oxygen species (ROS), while CO_2_ fixation is reduced, which is reflected in changes in contents of plant pigments such as chlorophylls and carotenoids^18^. The scavenging of ROS such as O_2_, H_2_O_2_ and OH is associated with the capability of *Salicornia* to manage salt stress effects. To mitigate oxidative damage, plants possess a complex antioxidant system, including peroxidase (POD, EC 1.17.1.7) and catalase (CAT, EC 1.11.1.6) among others enzymatic systems. POD is located in the apoplastic space and vacuoles^19^, where it catalyses the conversion of H_2_O_2_ to H_2_O and O_2_, while H_2_O_2_ is scavenged by CAT^20^.

All these chemical changes can influence the physical properties of plant cell walls, which are considered to be important physiological mechanisms of plant adaptation to salt stress^21^. Until now, there has been little knowledge of the role of cell anatomy, pectin content, biochemical parameters and expression genes when *S. europaea* populations from different maternal soils are subjected to salt stress.

According to Yadav et al.^22^, the *Salicornia* species uses different methods to adapt to salinity: (1) an active Na^+^ efflux; (2) through the Na^+^ stored in vacuoles; and (3) through Na^+^ inhibition, some antiporters play an important role to regulate the ion homeostasis in plants. For instance, Na^+^/H^+^ maintain an adequate ion content in the cytosol, which decreases cytotoxicity. These antiporters are present in tonoplasts and are in charge of pumping Na^+^ into the vacuole, while *SOS1,* located in the plasma membrane, pump Na^+^ into the apoplast^23^. Then, *S. europaea* cells can remain turgid with a continued proper cell function by ion compartmentalising in cell vacuoles. In this sense, the cytoplasm and organelles of the cells are protected from salinity.

Understanding the anatomical and biochemical responses of plants to salt stress and mining the salt tolerance associated with its maternal environment in nature are important for us in order to identify the most salt-tolerant crops in future. In this study, we hypothesised that *S. europaea* from different maternal salinities (a natural high salinity of ~ 1000 mM and an anthropogenic lower salinity of ~ 550 mM) can adapt differently to salt stress. Therefore, the aim of the present study was to investigate the influence of the maternal habitat in the salt tolerance of two different populations of *S. europaea* by evaluating the anatomical changes of stem-cortex cells from the fresh-water-storing tissue of plants subjected to saline stress conditions, and their correlation with changes in biochemical parameters and in the expression of *NHX1* and *SOS1* genes involved in sodium stem segregation. Principal component and Pearson analyses were used to identify the multiple responses of the two *S. europaea* populations in order to determine which one is the more sensitive to salinity.

## Results

### Morphometrical parameters in salinity gradient

**Figure 1:**
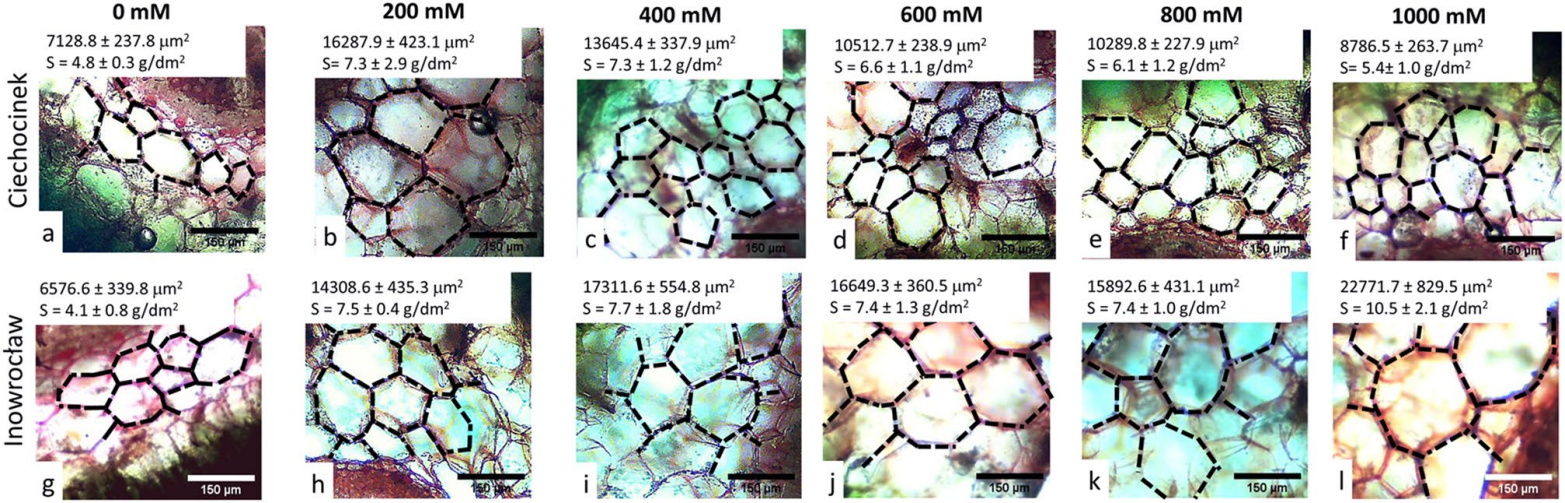
Stem-cortex cell’s area changes of *S. europaea* after 2 months in Cie (**a–f**) and Inw (**g–l**) populations grown under different NaCl concentrations. Scale bar 150 μm. n = 300 ± 50 cells, 12 individuals per treatment. S: correspond to the degree of succulence in the stems^17,24^. The *F* value of S that corresponds to the 2-way ANOVA for the interaction salt treatment × population is *F*_5,48_ = 5.5; *p* < 0.001.

**Figure 2:**
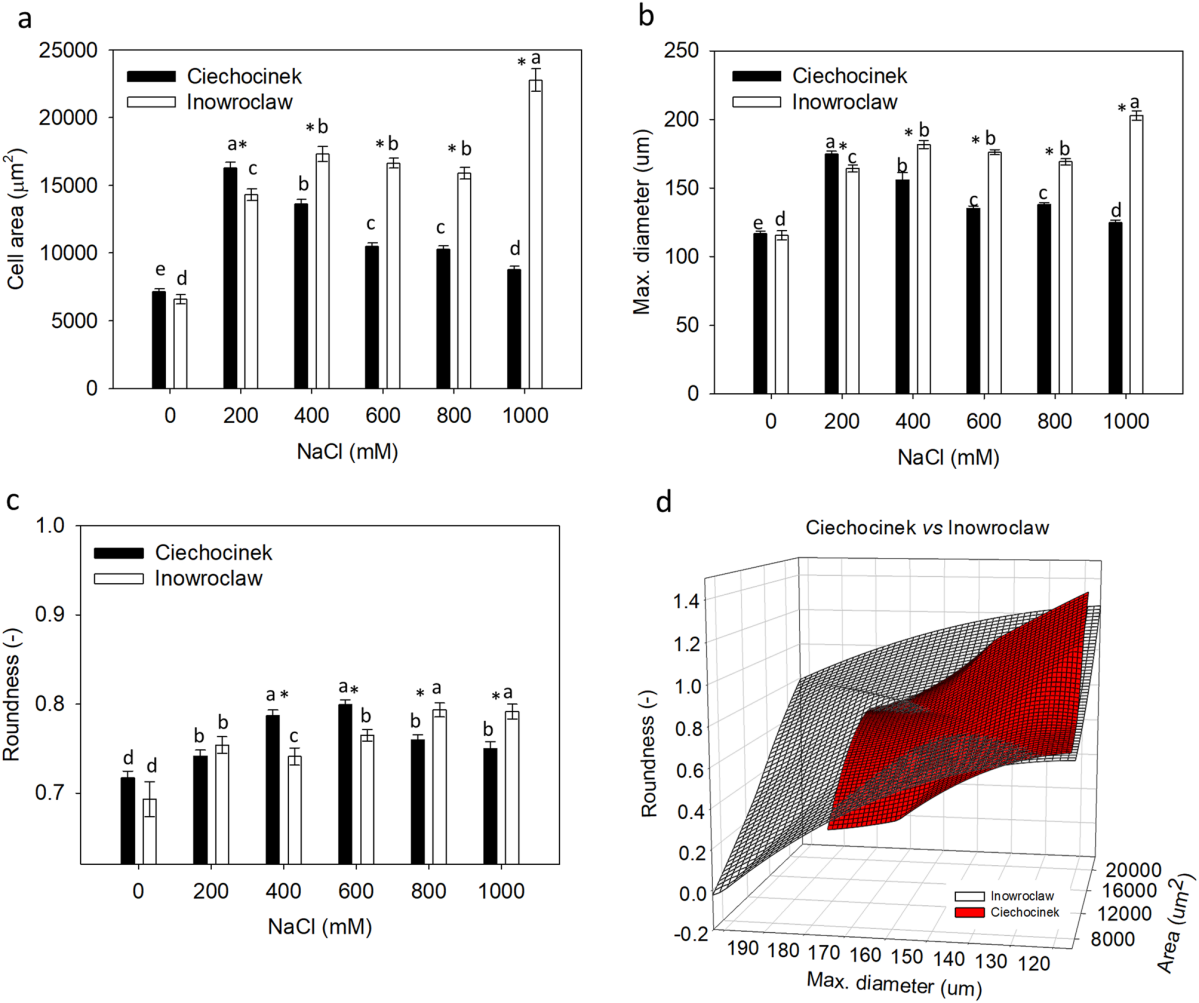
Morphometrical parameters (**a**) cell’s area, (**b**) maximum diameter, (**c**) roundness of the water storage cells in *S. europaea* populations (Inowrocław and Ciechocinek) under NaCl stress. (**d**) 3D plot compiling the three morphometrical parameters through the 6 salinity treatments. Means and ± SE of replicates. Different letters indicate significant differences between treatments within population and * indicates significant difference between populations within treatment (*p* < 0.05), n = 300 ± 50 cells, 12 individuals per treatment. The *F* values that correspond to the 2-way ANOVA for the interaction of salt treatment × population are *F*_5,288_ = 87.2, *F*_5,288_ = 89.9, and *F*_5,288_ = 9.7 for area, max. diameter and roundness respectively with a *p* < 0.001.

Overall, both populations showed different behaviour in terms of the morphometric parameters of the water storage cells of *S. europaea* plants from two different populations. The results obtained demonstrated significant differences in cell’s area (A) for all salt treatments, except for 0 mM (Fig. 1). Image analysis (IA) was useful for quantitatively characterising the self-similitude properties of plant anatomy, with the maximum cell’s area reached at 200 mM NaCl for Cie and at 1000 mM NaCl for Inw. Both populations showed significantly different A from treatment 200 to 1000 mM NaCl, at 0 mM both populations showed their minimum A (Figs. 1 and 2a). Cie has its maximum A at 200 mM with a gradual decrease along with salinity gradient, while for Inw, an increase was observed through all the treatments. An increase of 128% and 246% was observed between the minimum and maximum A for Cie and Inw, respectively. Between populations, A showed the highest difference between Cie and Inw at 1000 mM NaCl, with an increase of 159% in Inw with respect to Cie. The degree of succulence (S) of the stems was also calculated, these results adequately show the change between salt treatments, the values are in accordance to Delf^17^ report. The highest S is observed for Inw population at 1000 mM.

Then, a maximum increase in the cell’s diameter (Cdiam) and roundness (R) of water-storing cells was observed from 0 to 1000 mM NaCl, for Inw population, 75.5%, 11.3% respectively, while Cie population, has its maximum Cdiam 49.7% increase from 0 to 200 mM NaCl treatments, and R increases 11.5% from 0 to 600 mM NaCl (Fig. 2b,c). Therefore, a significant different behaviour from Cie and Inw population was detected as shown in 3D plot (Fig. 2d) which comprises the three morphological parameters of both populations through the 6 salinity treatments. Inw population showed a wider distribution suggesting a better adaptation during the experimental salinity stress with respect to Cie which presents a reduced distribution in the 3D plot.

### Biochemical modification to salt stress

**Figure 3:**
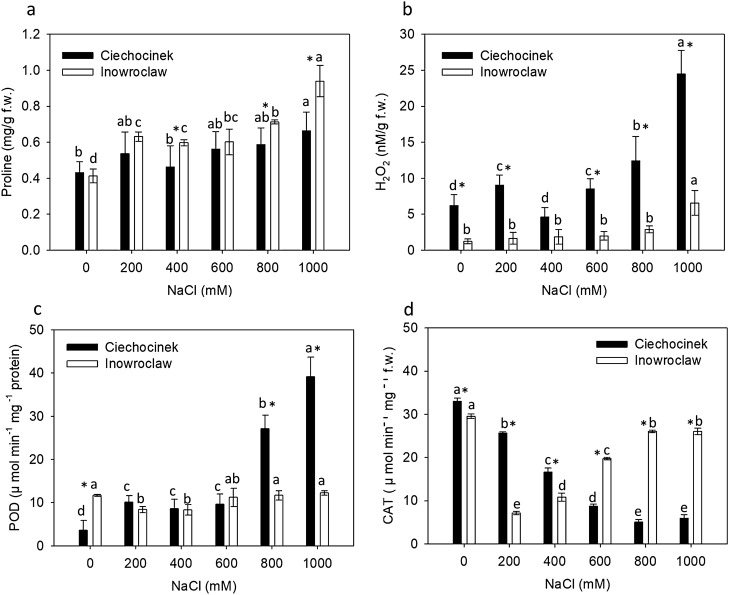
(**a**) Proline, (**b**) hydrogen peroxide contents, and specific enzyme activities of (**c**) POD and (**d**) CAT in *S. europaea* populations (Inowrocław and Ciechocinek) under NaCl stress. Means and ± SD of replicates. Different letters indicate significant differences between treatments within population and * indicates significant difference between populations within treatment (*p* < 0.05), n = 3. The *F* values that correspond to the 2-way ANOVA for the interaction of salt treatment × population are *F*_5,24_ = 2.3, *F*_5,24_ = 8.1, *F*_5,24_ = 54.5 and *F*_5,24_ = 35.9 for proline, hydrogen peroxide, POD and CAT respectively with a *p* < 0.001.

Proline (P) showed an increase with salinity gradient (Fig. 3a). The results show that P was significantly higher in Inw in comparison to the Cie population under salt stress, mainly at 400, 800 and 1000 mM. Meanwhile, hydrogen peroxide (HP) was significantly higher in Cie with respect to Inw population through 0, 200, 600, 800 and 1000 Mm NaCl treatments. A significant increase was observed at 800 and 1000 mM NaCl in Cie and very slightly at 1000 mM NaCl in Inw population. Regarding the enzyme activity analysed, peroxidase (POD) activity increased markedly at 800 and 1000 mM NaCl in Cie with respect to Inw, which maintains a homogeneous low POD activity through all the treatments. These results correlate with the HP analysis (Fig. 3b,c). Then, the lowest catalase (CAT) activity for Inw was found at medium salinity treatments 200, 400, 600 mM, while the highest CAT activity was found at the extremes (0, 800 and 1000 mM), in contrast, for Cie population the CAT activity decreased along with the salinity gradient, both populations showed significant difference between them in all the treatments (Fig. 3d).

Chlorophyll *a* (Cha), *b* (Chb) and carotenoid (Carot), content show a remarkable decrease in both populations under NaCl stress (Table 1). The chlorophyll content among Inw and Cie was significantly different in Cha at 200 mM and in Chb at 0 and 200 mM. No significant differences between the two populations were found in total chlorophyll content, but in carotenoid, the highest content was found only at 0 mM for Cie and at 0 and 200 mM in Inw. However, comparing both populations, significant differences were observed through all treatments. Interestingly the total soluble protein content was higher for Cie at 0 mM treatment and it progressively decreases along with salinity gradient, while it increases with salinity in Inw.

**Table 1:** Changes in the chlorophylls *a*, *b*, total, carotenoid and total soluble protein content in *S. europaea* populations (Inowrocław and Ciechocinek) under NaCl stress. Means and ± SD of replicates. Different letters indicate significant differences between treatments within population and * indicates significant difference between populations within treatment (p < 0.05), n = 3. The F values correspond to the 2-way ANOVA interaction salt treatment × population.

Population	Treatment	Chlorophyll *a* (mg/g)	Chlorophyll *b* (mg/g)	Total chlorophyll (mg/g)	Carotenoids (mg/g)	Protein (mg/g)
Ciechocinek	0	0.72 ± 0.05a	0.22 ± 0.03a*	0.93 ± 0.08a	0.12 ± 0.01a*	6.62 ± 0.18a
	200	0.69 ± 0.01a*	0.13 ± 0.01b*	0.82 ± 0.02b	0.09 ± 0.003b*	5.62 ± 0.31ab
	400	0.32 ± 0.03b	0.10 ± 0.02b	0.43 ± 0.03c	0.05 ± 0.04c*	5.75 ± 0.14ab
	600	0.33 ± 0.04b	0.10 ± 0.04b	0.42 ± 0.07c	0.05 ± 0.03c*	4.61 ± 0.27b*
	800	0.35 ± 0.01b	0.09 ± 0.01b	0.43 ± 0.02c	0.05 ± 0.002c*	2.39 ± 0.31c*
	1000	0.24 ± 0.02c	0.09 ± 0.01b	0.32 ± 0.03d	0.04 ± 0.003c*	2.23 ± 0.01c*
Inowrocław	0	0.60 ± 0.09a	0.38 ± 0.06a*	0.99 ± 0.11a	0.24 ± 0.04a*	6.34 ± 0.46bc
	200	0.54 ± 0.01a*	0.25 ± 0.02b*	0.79 ± 0.03b	0.21 ± 0.01b*	5.09 ± 0.99c
	400	0.34 ± 0.04b	0.09 ± 0.02c	0.42 ± 0.08c	0.11 ± 0.03c*	6.61 ± 0.79bc
	600	0.32 ± 0.03b	0.08 ± 0.02c	0.40 ± 0.05c	0.09 ± 0.03c*	7.13 ± 0.28b*
	800	0.31 ± 0.04b	0.08 ± 0.01c	0.39 ± 0.05c	0.11 ± 0.02c*	7.11 ± 0.98b*
	1000	0.21 ± 0.02c	0.09 ± 0.02c	0.30 ± 0.04c	0.11 ± 0.02c*	9.05 ± 1.03a*
		F_5,24_ = 1.3; *p* = 0.20	F_5,24_ = 8.07; *p* < 0.001	F_5,24_ = 0.5; *p* = 0.67	F_5,24_ = 2.4; *p* = 0.08	F_5,24_ = 5.3; *p* = 0.00*2*

### High and low methylesterified HGs content and distribution under salt stress

**Figure 4:**
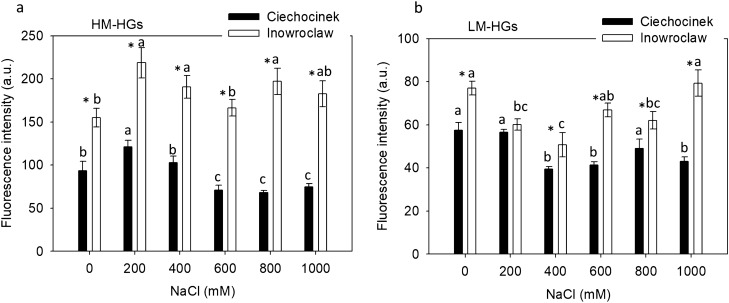
(**a**) High methylesterified homogalacturonans (HM-HGs) fluorescence intensity identified with JIM7 antibody; and (**b**) Low methylesterified fluorescence (LM-HGs) identified with LM19 antibody, in *S*. *europaea* stem cross-sections of Ciechocinek and Inowrocław populations subjected to 6 salinity treatments. Means and ± SD of replicates. Different letters indicate significant differences between treatments within population and * indicates significant difference between populations within treatment (p < 0.05), n = 3. The F values that correspond to the 2-way ANOVA for the interaction salt treatment × population are F_5,48_ = 3.6, p = 0.007 and F_5,48_ = 4.5, p = 0.002 for HM-HGs and LM-HGs respectively.

Immunofluorescence analysis of the location of high and low methylesterified HGs (HM-HGs and LM-HGs) showed variances in the total levels of methylesterified HGs as well as in their distribution through the semi-thin cross-section of the fleshy tissue among the stem, epidermis, palisade tissue, cortex, vascular bundles and vascular cylinder. An increase in the total intensity level of HM-HGs when subjected to salt stress was identified with JIM7 antibody in the stem cross-section for Inw, whereas for Cie the highest total intensity levels of HM-HGs were observed only at 200 mM NaCl, then a gradual decrease occurred along with the salinity gradient (Fig. 4a). LM-HGs homogalacturonans distribution identified with LM19 antibody show significantly higher levels of total intensity for Inw with respect to Cie in all salt treatments, with exception of treatment at 200 mM (Fig. 4b).

**Figure 5:**
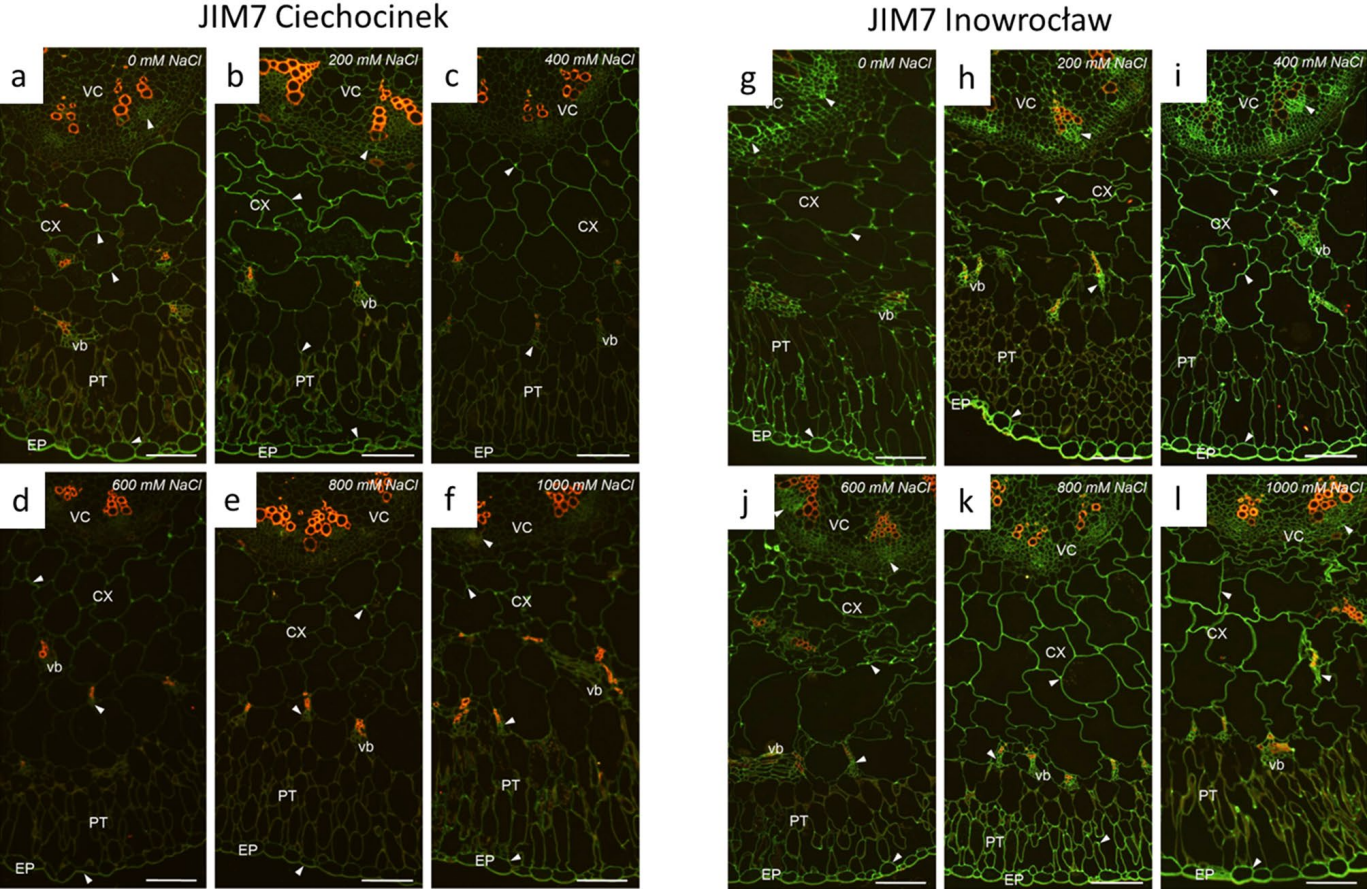
Immunofluorescence localization (arrowheads) of high methylesterified homogalacturonans (HM-HGs) under NaCl stress of two *S. europaea* populations. (**a**–**f**) Ciechocinek and (**g**–**l**) Inowrocław. *EP* epidermis, *PT* palisade tissue, *CX* cortex, *vb* vascular bundle, *VC* vascular cylinder. Scale bar 100 μm.

**Figure 6:**
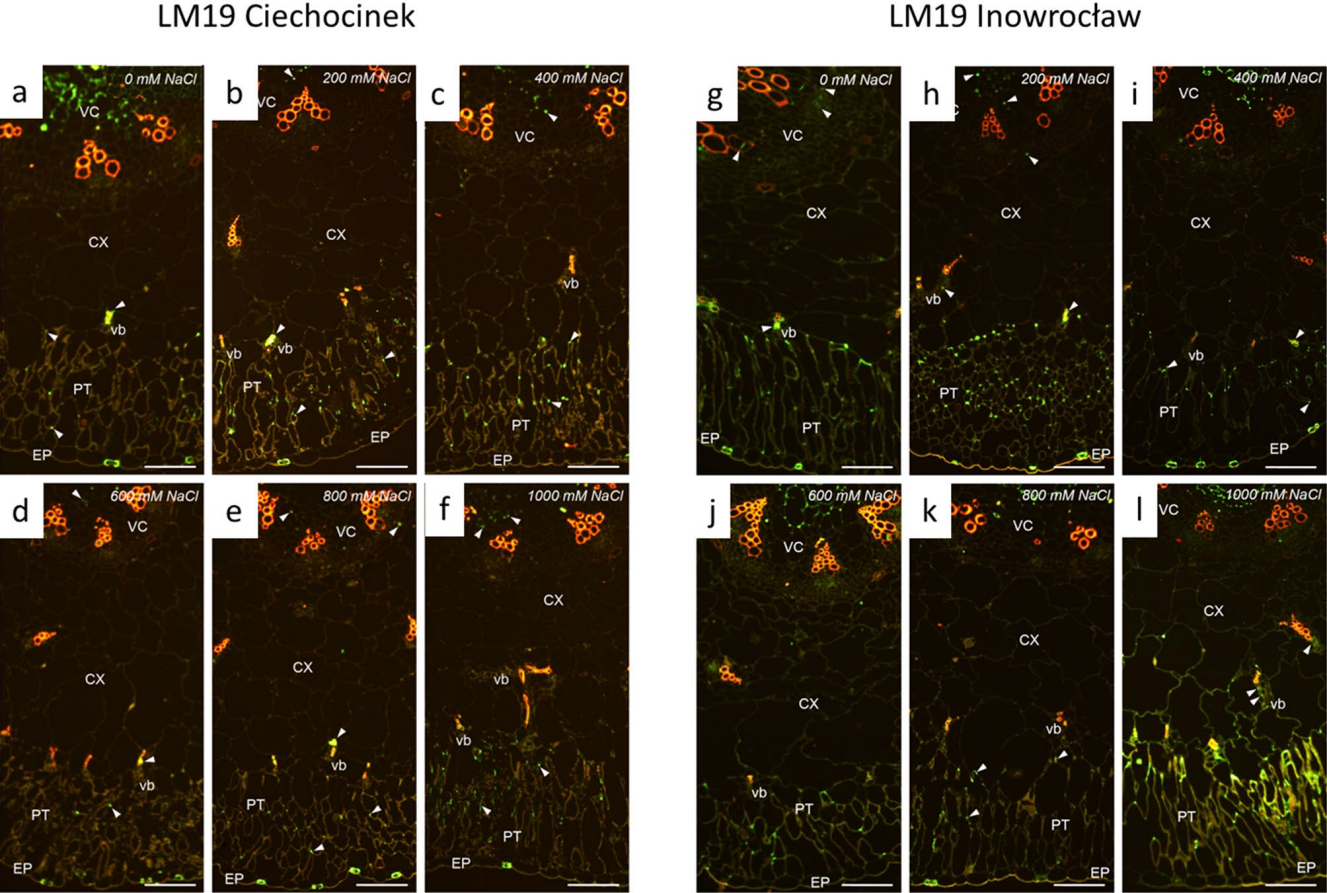
Immunofluorescence location (arrowheads) of low methylesterified homogalacturonans (LM-HGs) under NaCl stress for two *S. europaea* populations. (**a**–**f**) Ciechocinek and (**g**–**l**) Inowrocław. *EP* epidermis, *PT* palisade tissue, *CX* cortex, *vb* vascular bundle, *VC* vascular cylinder. Scale bar 100 μm.

Also, the HM-HGs and LM-HGs quantity varied between treatments and populations as observed in Fig. 5a–f (Ciechocinek) vs. Fig. 5g–l (Inowrocław) for HM-HGs and Fig. 6a–f (Ciechocinek) vs. Fig. 6g–l (Inowrocław) for LM-HGs, though the different anatomical sections, epidermis and vascular bundle tissue showed the highest content of fluorescence intensity of HM-HGs and LM-HGs in Table 2. In particular, epidermis tissue showed the highest content of HM-HGs and LM-HGs in both populations. However, the results indicate significantly higher content in Inw at 200 and 400 mM NaCl for HM-HGs and at 0 mM and 1000 mM NaCl for LM-HGs. Then, vascular bundle tissue was the section with the second-highest content of HM-HGs and LM-HGs for both populations, though differences were identified: for Cie, the highest contents for both HM-HGs and LM-HGs were found at 200 mM NaCl (Figs. 5b and 6b, arrowheads, respectively; Table 2); meanwhile, in Inw, HM-HGs showed high contents in the vascular bundle region, from 200 to 1000 (Fig. 5h–l, Table 2). For LM-HGs in Inw, non-significant differences were found between treatments (Fig. 6h–l arrowheads, respectively; Table 2). In the palisade tissue a significant increase in LM-HGs is identifiable at 1000 mM NaCl for the Inw population (Fig. 6l, Table 2).

**Table 2:** Immunofluorescence of *S. europaea* stem cross-section distribution and quantification of low and high methylesterified homogalacturonans (LM-HGs and HM-HGs) under NaCl stress for two populations (Ciechocinek and Inowrocław). Means and ± SD of replicates. Different letters indicate significant differences between treatments within population and * indicates significant difference between populations within treatment (p < 0.05), n = 5. The *F* values correspond to the 2-way ANOVA interaction of salt treatment × population.

Treatment	Epidermis (EP)		Palisade tissue (PT)		Cortex (CX)		Vascular bundle (vb)		Vascular cylinder (VC)	
	Cie	Inw	Cie	Inw	Cie	Inw	Cie	Inw	Cie	Inw
**HM-HGs (JIM7)**										
0	36.2 ± 1.13a*	56.9 ± 5.43c	13.1 ± 0.98b*	18.9 ± 1.99bc	10.1 ± 0.48b*	14.9 ± 1.99c	21.5 ± 5.03b*	37.9 ± 2.84c	12.6 ± 2.20b*	26.5 ± 2.53ab
200	37.2 ± 3.21a*	86.1 ± 9.70a	16.2 ± 0.76a*	28.7 ± 2.84a	13.2 ± 0.58a*	22.0 ± 2.84a	36.4 ± 3.53a*	54.1 ± 7.25ab	18.4 ± 1.07a*	27.7 ± 3.38ab
400	28.8 ± 3.33a*	74.2 ± 7.48ab	14.7 ± 2.84ab	16.6 ± 1.64c	10.9 ± 0.74b*	16.9 ± 1.64bc	31.4 ± 3.82a*	52.1 ± 4.05ab	17.1 ± 1.17a*	30.9 ± 2.06a
600	20.7 ± 2.89b*	60.6 ± 3.43bc	9.95 ± 0.83bc*	17.4 ± 0.98bc	7.5 ± 0.37b*	18.1 ± 0.98ba	21.9 ± 3.57b*	46.0 ± 5.90b	10.8 ± 0.74b*	24.5 ± 1.30b
800	16.4 ± 0.98b*	64.3 ± 5.89bc	9.61 ± 0.39bc*	26.2 ± 1.99a	7.8 ± 0.21ab*	19.2 ± 1.99ba	21.2 ± 1.96b*	55.51 ± 7.19ab	13.0 ± 0.69b*	31.9 ± 2.60a
1000	18.9 ± 1.16b*	55.4 ± 6.40c	11.1 ± 0.42bc*	21.3 ± 1.75b	8.5 ± 0.31b*	17.3 ± 1.79bc	23.6 ± 2.46b*	59.6 ± 9.63a	12.5 ± 0.21b*	28.9 ± 1.44a
	*F*_5,48_ = 2.1;* p* = 0.005		F_5,48_ = 6.2; p < 0.001		F_5,48_ = 1.7; p = 0.14		F_5,48_ = 2.2; p = 0.06		F_5,48_ = 1.9; p = 0.09	
**LM-HGs (LM19)**										
0	11.9 ± 1.13a*	23.2 ± 2.43a	9.486 ± 0.53a*	12.8 ± 0.45b	5.7 ± 0.28a*	7.5 ± 0.33a	19.3 ± 3.84a	22.2 ± 2.21a	11.1 ± 0.43a	11.3 ± 0.61a
200	11.3 ± 1.15a*	16.8 ± 1.731b	9.541 ± 0.42a	9.7 ± 0.89c	5.6 ± 0.26a	5.4 ± 1.0c	20.6 ± 3.04a	19.6 ± 0.98a	9.6 ± 0.55b	8.8 ± 0.50bc
400	9.6 ± 0.81ab*	17.7 ± 3.93b	7.17 ± 1.32b	7.3 ± 0.67d	4.3 ± 0.08b	4.9 ± 0.92c	11.5 ± 1.19c	12.8 ± 1.65b	6.9 ± 0.22c	7.9 ± 0.62c
600	10.8 ± 1.05a*	13.6 ± 0.89c	7.252 ± 1.42b*	12.4 ± 1.16b	3.9 ± 0.72b*	6.8 ± 0.17b	12.5 ± 0.77c*	22.6 ± 3.16a	6.8 ± 0.15c*	11.5 ± 0.62a
800	12.3 ± 2.87a*	17.8 ± 1.79b	7.450 ± 1.39b*	9.7 ± 0.77c	4.2 ± 0.14b*	5.2 ± 0.97c	17.4 ± 1.84ab	19.8 ± 2.42a	7.6 ± 0.16bc*	9.5 ± 0.80b
1000	9.17 ± 1.18b*	19.5 ± 3.42ab	7.870 ± 1.49b*	16.5 ± 1.66a	4.9 ± 0.95ab*	8.02 ± 0.45a	15.3 ± 1.99b*	22.9 ± 3.09a	5.9 ± 0.34c*	12.5 ± 1.28a
	*F*_5,48_ = 2.3;* p* = 0.02		*F*_5,48_ = 9.3; *p* < 0.001		*F*_5,48_ = 14.3; *p* < 0.001		*F*_5,48_ = 1.7;* p* = 0.13		*F*_5,48_ = 11.1; *p* < 0.001	

### Evaluation of the differences between *S. europaea* populations

**Figure 7:**
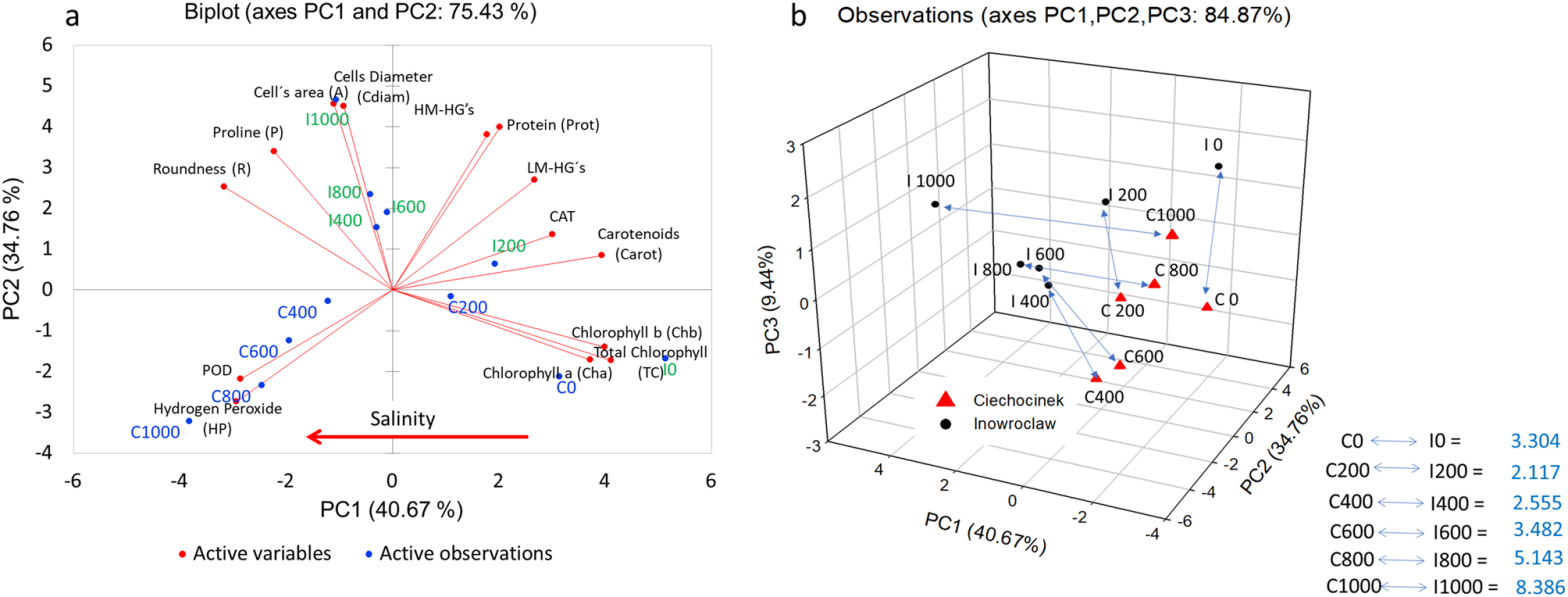
(**a**) Bi-plot of the first two principal components with all variables and observations, showing distribution of samples along the gradient of salinity going from right to left. (**b**) Three main principal components represented in a 3D plot showing distances per treatment among both populations. *Inw* Inowrocław, *Cie* Ciechocinek, 0, 200, 400, 600, 800 and 1000 indicate the concentrations in mM of NaCl, and PC the corresponding principal component.

All the variables were evaluated in each population using principal component analysis (PCA) (Fig. 7a); both populations show a similar tendency at the low salt treatments. Figure 7a shows the PC1 and PC2 axes, which accurately describe the variance of the samples (75.43%). This plot shows which plants are the most tolerant with regard to salt stress and how they correlate with the active variables that describe the low or high stress. It also shows that the Inw population seems to cope better with salinity. The biplot demonstrates that I1000 mM correlates well with the cell area variable, which is the morphometric trait that suggests Inw is less affected under stress salinity; this agrees with the image growth analysis reported in a previous salinity tolerance study for the same populations^25^. Variables related to high stress, such as HP and POD, correlate better with high salinity treatments in Cie (Fig. 7a). This biplot also shows how the individuals move through the two-dimensional space of the main components, from the positive to the negative quadrant of PC1 as salinity increases. The results were also grouped on a 3D plot (Fig. 7b) according to their similarities through the three principal component scores (PC1, PC2 and PC3) that describe the variance of the samples (84.87%), which shows that Cie plants are more susceptible to salt stress. Factorial scores from the PCA of each sample were used to calculate the distance between the two points under the same treatment P1 = (x_1_, y_1_, z_1_) and P2 = (x_2_, y_2_, z_2_) in the 3D space of the PCA (Fig. 7b). The comparisons of C0 vs. I0 (3.30) against C1000 vs. I1000 (8.39) were created in the 3D cartesian axis (x = PC1, y = PC2, z = PC3), with distance results indicating that the greater the stress, the greater the separation. In addition, the shortest distance C200 vs. I200 (2.12) is observed at the optimum salinity for *S. europaea*-growth, at between 200 and 400 mM NaCl.

### Expression patterns of *SeNHX1* and *SeSOS1* genes involved in Na^+^ segregation of *S. europaea* stem

**Figure 8:**
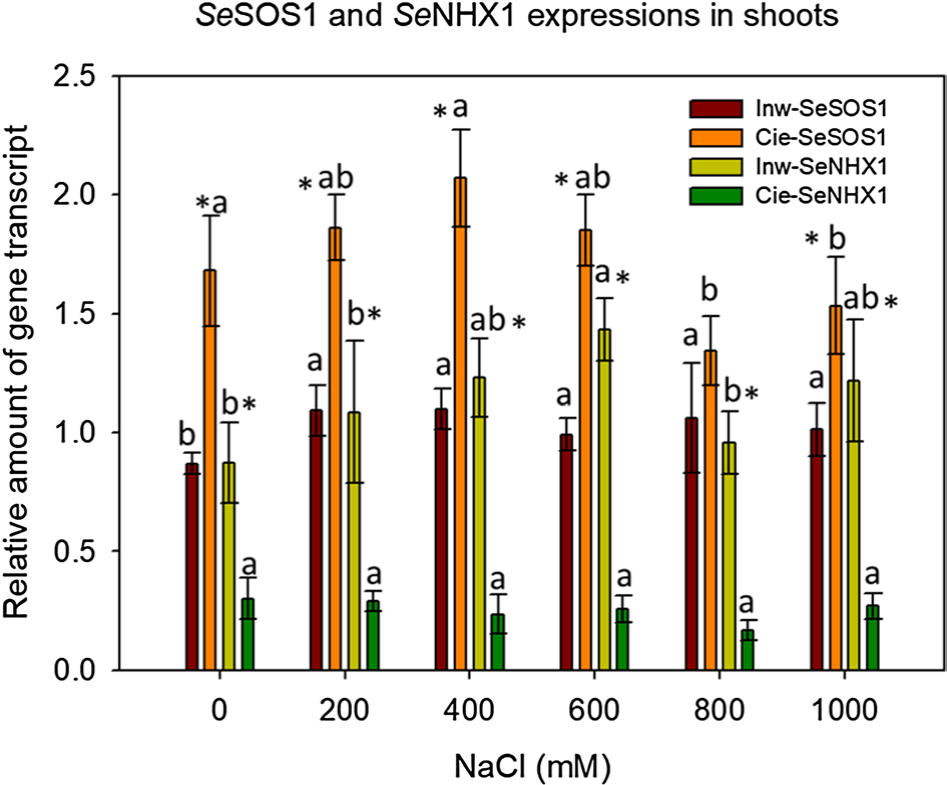
Expression analysis of *Se*NHX1 and *SeSOS1* genes involved in sodium segregation in shoots of *S. europaea* under different NaCl concentrations. Means and ± SD of replicates. Different letters indicate significant differences between treatments within population and * indicates significant difference between populations within treatment (p < 0.05), n = 3. The *F* values that correspond to the 2-way ANOVA for the interaction salt treatment × population are *F*_5,24_ = 2.7; *p* = 0.03 and *F*_5,24_ = 3.6; *p* = 0.01 for *Se*NHX1 and *SeSOS1* genes respectively.

The expression patterns of *NHX1* and *SOS1* in *S. europaea* stems under saline treatments were analysed with real-time quantitative reverse transcriptase polymerase chain reaction (qRT-PCR). These genes *NHX1* and *SOS1* encode a tonoplast Na^+^/H^+^ antiporter and apoplast antiporter, respectively. *SeNHX1* and *SeSOS1* expression does not show a significant difference within treatments of the same population, but a significant difference in gene expression is visible between populations. *SeNHX1* and *SeSOS1* were equally expressed in the Inw population, while the Cie population showed the highest expression for *SeSOS1* but very low *SeNHX1* expression, as shown in Fig. 8. This confirms differences between these two populations.

## Discussion

According to the *S. europaea* anatomical cells results obtained through image analysis, cell’s area has a similar result under 0 mM NaCl in both populations, but significant differences were observed when populations were subjected to salt stress. The Inw population has the highest values for all cell parameters tested. The highest value observed was in Inw at 1000 NaCl. Our results are in accordance with Akcin et al.^26^, who demonstrated that *Salicornia freitagii* stem anatomical characters such as thickness, length and width of water-storing tissue significantly increased when the halophyte grows under high salinity. While the roundness of the cells analysed in this study show that at higher salinities, cells lose their natural hexagonal shape, therefore, this parameter was useful to determine that cells turn round probably due to the high-water storage within them. The highest roundness was observed for Cie at 400 and 600 mM and for Inw population at 800 and 1000 mM suggesting that these rounded cells store higher amount of water. These parameters, area of cells and roundness, can be associated with an increase in (S) succulence as a way to aid in storing additional water by increasing vacuolar volume for better survival under harsh saline environment, as stated by Akcin et al., Debez et al. and Hameed et al.^26–29^, who also showed that succulence is an adaptation mechanism in salt-tolerant cultivars subjected to saline stress. Results of succulence in the present work, adequately show the change between salt treatments which are associated to the area of the cells, large cells can be linked to high turgidity and hence to the S of the plant. In the same line, proline, which allows additional water to be reserved in the water storage cells from the environment, positively correlates with the anatomical analysis. The 22% and 40% higher results for P in the Inw population at 800 and 1000 mM NaCl, respectively, relative to the Cie population can be linked to the increased cell area and roundness in the Inw population. These features may allow cell water potentials to decrease^30,31^. Kumar et al.^31^ demonstrated that between two cultivars of *Morus alba *L*.* subjected to salt stress, the proline metabolism was significantly altered and the extent of alteration varied between both cultivars, where proline accumulation was higher in the salt tolerant cultivar than in the salt sensitive one because higher content of proline leads to the maintenance of turgor by preventing the loss of water and ion toxicity, supporting its salt tolerance. Also, our results are in accordance with studies carried out by Aghaleh et al. and Akcin and Yalcin^30, 32^ for *S. europaea*. Moreover, the Cie population showed the highest HP and POD values, especially at the highest salinity, with percentage differences of 285% and 219%, respectively, with respect to Inw; in the Fig. 3b,c we can determine which population is more salt-tolerant. According to Kong and Seo^33^, salt-tolerant cultivars showed less HP content compared to salt-sensitive cultivars, due to the effect of salinity induction of reactive oxygen species (ROS), such as HP, which severely reduced overall plant growth in sensitive species. In the present study, the results indicate that the Cie population is more salt-sensitive than is the Inw population. Aghaleh et al.^32^ tested the effects of salt stress on the activities of antioxidative enzymes in two *Salicornia* species at NaCl concentrations (0, 100, 200, and 300 mM), finding that the salinity progressively enhanced the POD activity, whereas the CAT activity was only registered at the low salinity. POD and CAT play a key role in removing ROS produced in plant cells under abiotic stresses. In this study, the Cie population showed higher levels of POD activity under high salinity, probably due to the remarkably higher content of HP that this population has under high salinity with respect to Inw. Meanwhile, the decrease in photosynthesis activity when plants are subjected to salinity is reflected in the reduction of chlorophyll and CO_2_ fixation due to the lower stomatal conductance^27,32^. Some plants grown under high salinity have a lower stomatal conductance as a strategy to conserve water^34,35^. Consequently, CO_2_ fixation is reduced and photosynthetic rate decreases. The chlorophyll content of both populations was significantly different at 200 mM NaCl (Table 1), with no difference at high salinity. In this line, it is important to note that Chb type is an adaptive feature of adapted chloroplasts, because high Chb content produces an increase in the range of wavelengths absorbed by the chloroplasts, which is attributed as a mode of adaptation when plants are subjected to some abiotic stressor^36^. In the present study, Inw showed a statistically significant higher Chb content compared to Cie under 0 and 200 mM treatments, while Inw was the one with higher Carot content as a sign of a better adaptability to salt stress. The lower content of protein in the Cie population under salt stress and higher content in Inw population, suggests the possible connection of protein with an osmotic adjustment that confers higher salt tolerance. The importance of protein for abiotic and biotic stress adaptation was thoughtfully reviewed by Sasidharan et al.^37^, who stated that the regulation of cell wall protein activity results in growth modulation during stress, and that this can be mediated by the regulation of wall modifying proteins that alter cell wall structure and allow it to yield to turgor, thus driving a cellular expansion, which was corroborated with the cell area analysed in this study for each population. According to Zagorchev et al.^12^, around 30 kDa proteins are involved in the cell wall rigidity, which plays a crucial role in plant growth and development during stress adaptation. With regard to high and low methylesterified HGs, levels and distribution were noticeably different in each population. As pectin is important for the cell wall structure and could be modified in response to different signals such as salt stress, analysis of pectins received major attention in the present study. Overall, it is already known that a large majority of the genes encoding proteins modifying cell wall structure, are down- or up-regulated under salt treatment. In the case of pectin, Fan et al.^38^ reported that genes encoding methylesterases inhibitor family proteins are up-regulated under saline conditions, which decreases the level of methyl esterification of pectins and affects their normal function by inhibiting pectin methylesterase activity. This behaviour was reflected in the present study for Cie, the less salt-tolerant population. However, differences in the content of HM-HGs may occur between salt-tolerant populations. For instance, Uddin et al.^39^ indicate that under stress conditions the concentration of methylated pectic epitopes tends to drop, especially for those species that are less tolerant to salt stress. Meanwhile, Liu et al.^40^ indicate that the degree and pattern of the methyl-esterification of pectin to some extent determines the stiffness of cell walls and, with this, the tolerance to salinity. In the same study it is stated that the overexpression of the gene (*AtPMEI13*) that causes a decrease in pectin methylesterified enzyme activity in *Arabidopsis* enhances the total levels of methyl-esterification pectins, which was reflected in an improvement in seed germination and survival growth rate under salt stress. Also, Le Gall et al.^11^ reported that in salt-sensitive species, the high salinity triggers the de-esterification of loosely bound pectins that impede the swelling of cells, affecting the plant more than those with higher tolerance. According to Peaucelle et al.^15^, the de-esterification of homogalacturonans can lead to cell wall stiffening through the creation of “egg boxes”, and to enzymatic degradation of pectin, which indicates a denser and less extensible cell wall. On the other hand, HM-HGs can be involved in remodelling the cell wall structure and mechanical properties, which under salt stress helps to regulate the cell wall elongation and cell shape for better water accumulation, which translates into higher resistance to abiotic factors such as salinity^11^, as confirmed in the present study.

Herein, high methylesterified pectin was detected at high levels mainly in epidermis (ep) and in vascular bundles (vb) (Table 2). This vb tissue corresponds to the collenchyma cells, which are elongated cells composed of cellulose and pectin, with irregularly thick cell walls that provide support and structure. These cells are often found under the epidermis and associated with vascular bundles. A study carried out on three maize hybrids with contrasting salt tolerances showed an accumulation of highly methylated pectin in the salt-tolerant maize genotype, which favoured their cells’ elongation^39^. Another study reported by Muszyńska et al.^2^ showed that high methylated pectin (identified by immunolabelling with JIM7 antibody) increased within the cell wall of *Populus tremula* under saline conditions. This increase was linked with a rise in the modulus of elasticity and a decrease in cell wall plasticity in order to keep the turgor pressure necessary for plant growth. According to these authors, under salt stress, cell walls of salt sensitive cultivars can became more rigid (less flexible) while turgor pressure is maintained. Thus, maintaining good cell wall flexibility might be part of the mechanism by which salt-tolerant cultivars adapt to environmental stresses. Pectin polysaccharides are also believed to play an important role in cell adhesion and tissue cohesion^41^, which would be very important for adaptation to stress in plants that live in saline environments. For instance, in the halophyte *Sonneratia alba* a decrease in calcium content was detected, which may be a strategy by this halophyte to reduce cell rigidity^42^. Meanwhile, Le Gall et al.^11^ reported that, in *Salicornia europaea*, the genes encoding the cell wall proteins of the primary cell wall (including UDP-l-rhamnose synthase and cellulose synthases) decrease under saline conditions, while other genes that encode pectin methylesterase inhibitor proteins increase. Byrt et al.^43^ reported that there are associations between higher cell wall pectin content and increased tolerance to salinity.

According to Rasouli et al.^44^, salinity altered the physical properties of epidermic cells, specifically in the guard cell wall. In their study they demonstrated that the cell-wall-modifying enzymes such as acetyl- and methyl-esterifications esterases of pectin were upregulated in the epidermic cells of the halophyte *Chenopodium quinoa.* They concluded that the methyl-esterifications of pectins at epidermis are critical for salt tolerance by increasing the mechanical strength in the guard cells that are exposed to salinity. So, pectin methyl-esterification is essential for plant responses to environment stresses, which was also observed in the present study through the quantification of the fluorescence of the high methyl esterified pectin. The Inw population had a higher level of HM-HGs in the epidermis cells than did Cie, through all the salt treatments. This finding may also be associated with the fact that pectins in guard cell walls provide strength and flexibility in order to accommodate the turgor-pressure-driven changes in size and shape that underlie the opening and closing of stomatal pores during abiotic stress factors^45^. Moreover, for the case of vascular bundles, Fan et al.^38^ reported that under salinity the *S. europaea* genes involved in cell wall metabolism are well linked with the vessel differentiation increment in xylem. In this sense, highly methyl-esterified pectin together with lignin in xylem are the main passage for assimilation of water and mineral elements^46^. So, the accumulation of large amounts of salt in *S. europaea* shoots under salinity requires a more rigid support transport system from root to shoot, which may be an important strategy for this halophyte when adapting to salinity. This may explain our results with regard to the higher levels of HM-HGs in the vb for Inw population. The results of the correlation between investigated parameters are of great interest and some have not been reported before, especially the positive correlation between proline and cell area (0.728) (Table 3); this result confirms that the higher the cell’s area, the higher the proline content, which promotes plant succulence. Roundness has similar correlation tendency with proline (0.672) due to cell turgidity, while the plants under 0 mM were mainly described by higher total content of chlorophyll (Fig. 7a), and these two variables (R and TC) have a high significant negative correlation (0.781).

**Table 3:** Pearson correlation matrix of the anatomical, biochemical and pectin content parameters.

Var	A	Cdiam	R	Prot	CAT	POD	HM-HGs	LM-HGs	P	HP	Cha	Chb	TC	Car
A	**1**													
Cdiam	**0.993**	**1**												
R	**0.577**	0.528	**1**											
Prot	**0.598**	**0.578**	0.129	**1**										
CAT	0.065	0.042	− 0.277	**0.722**	**1**									
POD	− 0.261	− 0.288	0.019	− **0.713**	− 0.528	**1**								
HM-HGs	**0.605**	**0.639**	0.028	**0.655**	0.227	**− **0.442	**1**							
LM-HGs	0.321	0.307	**− **0.219	**0.687**	**0.637**	**− **0.286	**0.628**	**1**						
P	**0.728**	**0.672**	**0.632**	0.289	**− **0.126	0.242	0.395	0.326	**1**					
HP	**− **0.316	**− **0.361	0.057	**− 0.709**	**− **0.419	**0.863**	**− 0.688**	**− **0.479	0.150	**1**				
Cha	**− **0.411	**− **0.363	**− 0.723**	0.052	0.482	**− **0.454	**− **0.009	0.187	**− 0.644**	**− **0.268	**1**			
Chb	**− **0.505	**− **0.482	**− 0.727**	0.097	0.379	**− **0.291	0.194	0.463	**− **0.494	**− **0.365	**0.701**	**1**		
TC	**− **0.476	**− **0.433	**− 0.781**	0.074	0.481	**− **0.427	0.068	0.307	**− 0.636**	**− **0.325	**0.964**	**0.866**	**1**	
Carot	**− **0.079	**− **0.039	**− **0.560	0.388	0.376	**− **0.423	**0.660**	**0.699**	**− **0.171	**− 0.628**	0.523	**0.856**	**0.688**	**1**

Values in bold are different from 0 with a significance level alpha = 0.05.

Moreover, the inverse correlation (− 0.688) between HM-HGs and HP is also an interesting finding, suggesting that when the plant is under salt stress, the chemical cell wall composition is restructured; this was observed in a reduction in pectin content along the salinity gradient. However, the more salt-tolerant population Inw increases its HM-HGs content, suggesting that this component is related to better salt resistance; furthermore, a significant positive correlation was detected between HM-HGs vs. A and Cdiam (0.605 and 0.639 respectively). Meanwhile, HP positively correlated with POD, as expected (0.863). The results in Fig. 7a illustrate population salt tolerance and how the two populations move through the biplot showing the active variables that correlate with the studied individuals depending on their salt tolerance. In the Inw population, individual I1000 correlates well with cell area and proline content, while in Cie, individuals C1000, C800 and C600 correlate well with HP and POD. Then, the C0 and I0 individuals correlate well with TC, Cha, Chb and Carot. Additionally, factorial scores (Fig. 7b) were useful in demonstrating that the highest separation between Inw and Cie parameters was found at the highest salinity, indicating also that Cie has more stress-modifications at this salinity level.

The present gene expression results confirmed that Inw can be considered a more salt-tolerant plant in comparison to the Cie population. This is because, according to Lv et al.^47^, *NHX1* is one of the Na^+^/H^+^ antiporters in tonoplast responsible for Na^+^ transport from cytosol to vacuole, and it plays a central role in salinity tolerance. In the present study, *SeNHX1* was highly expressed only in the Inw population, suggesting the important role of Na^+^/H^+^ in the Na^+^ influx to vacuole in plant cells. For instance, Hayatsu et al.^42^ reported that in the halophyte *Sonneratia alba*, the Na^+^ content in the vacuole was higher than in glycophyte *Oryza sativa,* concluding that halophilic cells gain salt tolerance by transporting Na^+^ into their vacuoles. Meanwhile, the *SeSOS1* gene was highly expressed in the salt-sensitive population (Cie), suggesting that excreting Na^+^ in apoplast is the main mechanism by which this population copes with salinity. Jha et al.^48,49^ stated that the transcript of a Na^+^/H^+^ antiporter gene from *Salicornia brachiata* (*SbNHX1*) increased under different NaCl concentrations. However, in the present study, no significant differences were found between salt treatments. This phenomenon may be attributed to the different plant species, salt treatments and experiment duration. Fan et al.^38^ found that more than half of the differentially expressed transcription factors in *Salicornia* are directly or indirectly involved in the salt response but also in the growth and development process of *S. europaea* roots and shoots. So, only a small fraction participated exclusively in the stress response, which means that salinity efficiently induces the growth of *S. europaea* and that most of these genes can be activated through all the development growth process, independently of the salt content.

All the discussed results confirm our hypothesis that different maternal salinity populations of the same *S. europaea* species adapt differently to salt stress at the anatomical, pectin, biochemical and gene level, which can be important in the context of *Salicornia europaea* species as future crop^50^, especially considering seed sources.

## Materials and methods

### Plant materials, growth conditions and salt treatments

Soil samples were performed as in previous experiments with *S. europaea*^25^, seeds were collected at two maternal sites, the first of which represents natural salinity related to inland salt springs at the health resort of Ciechocinek (Cie) (52°53′N, 18°47′E) characterised by a high soil salinity of *ca* 100 dS m^−1^ (~ 1000 mM NaCl), and the second of which is associated with soda factory waste that affects the local environment in Inowrocław-Mątwy (Inw) (52°48′N, 18°15′E) and with a lower salinity of *ca* 55 dS m^−1^ (~ 550 mM NaCl). The complete soil description is reported in Piernik et al.^51^ and Szymanska et al.^52,53^. Populations are isolated by a distance of *ca* 40 km without any saline environment between them, however, they were somehow connected due to the presence of salt springs in the nineteenth century. The seeds came from one generation and were collected in early November 2018. The seeds were germinated and grown according to the same steps reported in Cárdenas-Pérez et al.^25^ with a slight modification in the number of salt treatments at 0, 200, 400, 600, 800 and 1000 mM NaCl. In total, 144 plants were cultivated, and, therefore, a complete randomised factorial design 2^6^ was used, which included (12 plants × 6 treatments × 2 populations) with 14 response variables. After 2 months of development, anatomical analysis such as cell area (A), roundness (R) and maximum cell diameter (Cdiam) were estimated in 12 samples, whereas high and low methyl esterified pectins (HM-HGs and LM-HGs), proline (P), hydrogen peroxide (HP), total soluble protein (Prot), catalase activity (CAT), peroxidase activity (POD), chlorophyll *a*, *b* and total (Cha, Chb and TC), carotenoid (Carot) contents, as well as *SeNHX1* and *SeSOS1* gene expression, were all determined per triplicate (plants were randomly selected). The collection of plant material, comply with relevant institutional, national, and international guidelines and legislation, IUCN Policy Statement on Research Involving Species at Risk of Extinction and Convention on the Trade in Endangered Species of Wild Fauna and Flora. The voucher specimen of the plant material has been deposited in a publicly available herbarium of the Nicolaus Copernicus University in Toruń (Index Herbarium code TRN), deposition number not available (dr. hab. Agnieszka Piernik, prof. NCU undertook the formal identification of plant species, and permission to work with the seeds was provided by the Regional Director of Environmental Protection in Bydgoszcz, WOP.6400.12.2020.JC).

### Anatomical image analysis

From the middle primary branch (fleshy segment shoot) of *S. europaea* plant treatments (0, 200, 400, 600, 800 and 1000 mM NaCl), slices of fresh tissue were obtained by cutting them with a sharp bi-shave blade. The thinner slices of approximately 0.5 mm were selected and used in the microstructure analysis. The size and shape of the stem-cortex cells from the fresh water-storing tissue were characterised by a light microscope (Olympus BX51, USA) connected to a digital camera (DP72 digital microscope camera) and digital acquisition software (DP2-BSW). The microscope images were captured at a magnification of 10 ×/0.30 in RGB scale and stored in TIFF format at 1280 × 1024 pixels. A total of 300 ± 50 cells from five individuals per treatment were analysed. Finally, the shape and size of the cells were obtained from the captured images. Cell image analysis (IA) was performed in ImageJ v. 1.47 (National Institutes of Health, Bethesda, MD, USA). The following anatomical parameters were obtained. Firstly, the cell area (A) was estimated as the number of pixels within the boundary. Secondly, the maximum cell’s diameter (Cdiam) was determined by the distance between the two points separated by the largest coordinates in different orientations, and the cell roundness (R) was obtained through the equation R = (4 A)/(π (Cdiam)^2^)—where a perfectly round cell has R = 1.0, while elongated cells will show an R → 0. Finally, the degree of succulence (S) in stem was calculated according to^24^ with slight change S = (Fresh Weight-Dry Weight)/stem Area, where the Area of the stem (As) was calculated as: As = π × r^2^, the diameter of the stems was obtained according to Cárdenas-Pérez et al.^25^.

### Immunolocalisation experiments

The samples dissected from the middle segment of the shoot (3 individuals per treatment) were prepared for embedding in BMM resin (butyl methacrylate, methyl methacrylate, 0.5% benzoyl ethyl ether (Sigma) with 10 mM DDT (Thermo Fisher Scientific) according to Niedojadło et al.^54^. Next, specimens were cut on a Leica UCT ultramicrotome into serial semi-thin cross sections (1.5 µm) that were collected on Thermo Scientific Polysine adhesion microscope slides. Before immunocytochemical reaction, the resin was removed with two changes of acetone and washed in distilled water and PBS pH 7.2. After incubation with blocking solution containing 2% BSA (bovine serum albumin, Sigma) in PBS pH 7.2 for 30 min at room temperature, the sections were incubated with anti-pectin rat monoclonal primary antibody JIM7 (recognises partially methylesterified epitopes of homogalacturonan [HG] but does not bind to fully de-esterified HGs) or antibody LM19 (recognises partially methylesterified epitopes of HG and binds strongly to de-esterified HGs) (Plant Probes) diluted 1:50 in 0.2% BSA in PBS pH 7.2 overnight at 4 °C. After washing with PBS pH 7.2, the material was incubated with AlexaFluor 488-conjugated goat anti-rat secondary antibody (Thermo Fisher Scientific) diluted 1:1000 in 0.2% BSA in PBS pH 7.2 for 1 h at 37 °C. Finally, the sections were washed in PBS pH 7.2, dried at room temperature and covered with ProLongTMGold antifade reagent (Thermo Fisher Scientific). The control reactions were performed with the omission of incubation with primary antibodies. Semithin sections were analysed with an Olympus BX50 fluorescence microscope, with an UPlanFI 1009 (N.A. 1.3) oil immersion lens and narrow band filters (U-MNU, U-MNG). The results were recorded with an Olympus XC50 digital colour camera and CellB software (Olympus Soft Imaging Solutions GmbH, Germany).

### Fluorescence quantitative evaluation

For the quantitative measurement, each experiment was performed using consistent temperatures, incubation times and concentrations of antibodies. The aforementioned ImageJ (1.47v) software was used for image processing and analysis. The fluorescence intensity was measured for five semi-thin sections for each experimental population (Inowrocław and Ciechocinek) at the same magnification (100 ×) and the constant exposure time to ensure comparable results. The threshold fluorescence in the sample was established based on the autofluorescence of the control reaction. The level of signal intensity was expressed in arbitrary units (a.u.) as the mean intensity per μm^2^ according to Niedojadło et al.^54^.

### Biochemical analysis

Proline content (P) was measured according to Ábrahám et al.^55^. Five hundred milligrams of fresh stem material was minced on ice and homogenised with 3% aqueous sulfosalicylic acid solution (5 μl mg^−1^ fresh plant material), centrifuged at 18,000×*g*, 10 min at 4 °C, and the supernatant was collected. The reaction mixture: 100 μl of 3% sulphosalicylic acid, 200 μl of glacial acetic acid, 200 μl of acidic ninhydrin reagent and 100 μl of supernatant. Acidic ninhydrin reagent was prepared according to Bates et al.^56^. The standard curve for proline in the concentration range of 0 to 40 μg ml^−1^. The standard curve equation was y = 0.0467x − 0.0734, R^2^ = 0.963. P was expressed in mg of proline per gram of fresh weight. Hydrogen peroxide (HP) levels were determined according to the methods described by Velikova et al.^57^, and 500 mg of stem tissues were homogenised with 5 ml trichloroacetic acid 0.1% (w:v) in an ice bath. The homogenate was centrifuged (12,000×*g*, 4 °C, 15 min) and 0.5 ml of the supernatant was added to potassium phosphate buffer (0.5 ml) (10 mM, pH 7.0) and 2 ml of 1 M KI. The absorbance was read at 390 nm, and the HP content was given on a standard curve from 0 to 40 mM. The standard curve equation was y = 0.0188x + 0.046, R^2^ = 0.987. HP concentrations were expressed in nM per gram of fresh weight. Chlorophylls (Cha and Chb) and carotenoids were extracted from fresh plant stems (100 mg) using 80% acetone for 6 h in darkness, and then centrifuged at 10,000 rpm, 10 min. Supernatants were quantified spectrophotometrically. Absorbance was determined at 646, 663 and 470 nm and calculations were performed according to Lichtenthaler and Wellburn^58^, when 80% of acetone is used as dissolvent. Total chlorophyll content was calculated as the sum of chlorophyll *a* and *b* contents.

Total CAT activity was determined spectrophotometrically by following the decline in A_240_ as H_2_O_2_ (ε = 39.9 M^−1^ cm^−1^) was catabolised, according to the method of Beers and Sizer^59^. Decrease in absorbance of the reaction at 240 nm was recorded after every 20 s. One unit CAT was defined as an absorbance change of 0.01 units min^−1^. Total POD activity was determined spectrophotometrically by monitoring the formation of tetraguaiacol (ε = 26.6 mM^−1^ cm^−1^) from guaiacol at A_470_ in the presence of H_2_O_2_ by the method of Chance and Maehly^60^. Increase in absorbance of the reaction solution at 470 nm was recorded after every 20 s. One unit of POD activity was defined as an absorbance change of 0.01 units min^−1^. Total soluble protein (Prot) content was measured according to Bradford^61^ using bovine serum albumin (BSA) as a protein standard. Fresh leaf samples (1 g) were homogenised with 4 ml Na-phosphate buffer (pH 7.2) and then centrifuged at 4 °C. Supernatant and dye were pipetted in spectrophotometer cuvettes and absorbances were measured using a UV–vis spectrophotometer (PG instruments T80) at 595 nm^62^. Prot was determined based on the standard curve y = 1.6565x + 0.0837, R^2^ = 0.982, for total soluble protein in the concentration range of 0 to 1.2 mg ml^−1^ BSA. Triplicates per treatment were used for each analysis.

### Total RNA isolation

After 2 months of salt treatment, shoots of *S. europaea* plants (3 individuals per treatment) were washed several times with tap water and then three times with miliQ water. After drying, plant material was frozen in liquid nitrogen, and stored at − 80 °C. Total RNA isolation was performed using RNeasy Plant Mini Kit (Qiagen, Hilden, Germany) according to the manufacturer’s protocol. The quality and quantity of RNA was checked on 1.5% agarose gels in TAE (Tris–HCl, acetic acid, EDTA, pH 8.3) buffer stained with ethidium bromide, and by spectrophotometric measurement (NanoDrop Lite, Thermo Fisher Scientific, Waltham, MA, USA).

### Cloning of *SOS1* gene from *S. europaea* (*SeSOS1*)

One microgram (1 µg) of total RNA isolated from shoots of *S. europaea* was primed with 0.5 µg of oligo (dT)_20_ primer for 5 min at 70 °C. Then 4 µl of ImProm-II 5 × reaction buffer, 2 mM MgCl_2_, 0.5 mM each dNTP, 20 U of recombinant RNasin ribonuclease inhibitor, and 1 µl of ImProm-II reverse transcriptase (Promega, Madison, WI, USA) were added to a final volume of 20 µl. The reaction was performed at 42 °C for 60 min. To design degenerate primers for SOS1, cDNA sequences from *Arabidopsis thaliana* (NM_126259.4), *Lycopersicon esculentum* (AJ717346.1), *Mesembryanthemum crystallinum* (EF207776.1), *Oryza sativa* (AY785147.1), *Triticum aestivum* (AY326952.3), *Salicornia brachiata* (EU879059.1) were obtained from NCBI GeneBank. The sequences were aligned using the Clustal Omega tool (https://www.ebi.ac.uk/Tools/msa/clustalo/) and three pairs of degenerate primes were designed (listed in Table 4). The PCR reaction mixture includes cDNA, 0.2 µM each primer, 0.2 mM each dNTP, 4 µl of 5 × HF buffer, and 0.5 U of Phusion High-Fidelity DNA polymerase (Thermo Fisher Scientific, Waltham, MA, USA) in a total volume of 20 µl. The thermal conditions were as follows: 98 °C for 30 s, 98 °C for 10 s, gradient between 48 °C and 56 °C for 20 s, 72 °C for 60 s, 32 cycle, final extension for 10 min at 72 °C. A pair of primers deg2_F and deg2_R yielded a PCR product with expected size. The PCR product was purified from agarose gel, cloned into pJET1.2 vector (Thermo Fisher Scientific, Waltham, MA, USA) according to manufacturer’s protocol and sequenced (Genomed, Warsaw, Poland). The obtained partial cDNA sequence was named *SeSOS1* and deposited in NCBI GeneBank (acc. no. MZ707082).

**Table 4:** Sequences of the primers used for cloning of *SeSOS1* and quantitative real-time PCR.

Gene	Primer	Sequence 5′–3′	Amplicon size (bp)	References
*SeSOS1* degenerate primers	deg1_F	ACGCBGTBATCTTCKTCGGART	ca. 1800	This study
	deg1_R	AGAAADGCAGCACABATRARC		
	deg2_F	GNGGTACTMGVGTNCCCTAYACTG	ca. 1300	
	deg2_R	CCTAGYTCYTCRTCDTCHCGAAGATC		
	deg3_F	TCAATGGAARTTCAYCARATAAAG	ca. 1500	
	deg3_R	GCWGCTTGMACACCATTYARVARSCG		
*SeNHX1*	Forward	GGAGAATCGTTGGATGAATGAGTC	182	^64^
	Reverse	GCTTCTTTTTTACCTGGAACCCTG		
*SeSOS1*	Forward	GCGGTTGGAGTTTTGTTCCC	110	This study
	Reverse	AGGGATAATGCCACAGCTCC		
*SeCAC*	Forward	CGTGCCTTCTGATGCGACTA	166	^63^
	Reverse	TGCCTCTTCACTTGTGATGCT		

### Reverse transcription reaction and quantitative real-time PCR (qPCR) *SeNHX1* and *SeSOS1* gene expression analysis

Prior to reverse transcription reaction, RNA was treated with DNaseI (Thermo Fisher Scientific, Waltham, MA, USA). The cDNA was synthesised from 1.5 µg of total RNA using a mixture of 2.5 µM oligo(dT)_20_ primer and 0.2 µg of random hexamers with NG dART RT Kit (Eurx, Gdańsk, Poland) according to the manufacturer’s protocol. The reaction was performed at 25 °C for 10 min, followed by 50 min at 50 °C. The cDNA was stored at − 20 °C.

The PCR reaction mixture includes 4 µl of 1/20 diluted cDNA, 0.5 µM gene-specific primers (Table 4) and 5 µl of LightCycler 480 SYBR Green I Master (Roche, Penzberg, Germany) in a total volume of 10 µl. Clathrin adaptor complexes (CAC) was used as a reference gene^63^. The reaction was performed in triplicate (technical replicates) in LightCycler 480 Instrument II (Roche, Penzberg, Germany). The thermal cycling conditions were as follows: 95 °C for 5 min, 95 °C for 10 s, 60 °C for 20 s, 72 °C for 20 s, 40 cycles. The SYBR Green I fluorescence signal was recorded at the end of the extension step in each cycle. The specificity of the assay was confirmed by the melt curve analysis i.e., increasing the temperature from 55 to 95 °C at a ramp rate 0.11 °C/s. The fold-change in gene expression was calculated using LightCycler 480 Software release 1.5.1.62 (Roche, Penzberg, Germany).

### Statistical and multivariate analysis

In order to determine the projection of the effect of salt treatment in plants we followed Cárdenas-Pérez et al.^25^ methodology. A principal component analysis (PCA) was developed using XLSTAT software version 2019.4.1^65^. For this analysis, 14 variables were used, (A, Cdiam, R, Prot, CAT, POD, HM-HGs, LM-HGs, P, HP, Cha, Chb, TC, Carot), arranged in a matrix with the average values obtained from replicates of each treatment and population. A two-way ANOVA comparing treatments within populations and populations within treatments was conducted for all the results with the Holm–Sidak method. The data was fit with a modified three parameter exponential decay using SigmaPlot version 11.0^66^. The relationships between variables were performed using a Pearson analysis, while a significance test (Kaisere Meyere Olkin) was performed in order to determine which variables had a significant correlation with each other (α = 0.05). Then, a 3D plot was developed using the three principal component factors according to the Kaiser criterion which stated that the factors below the unit are irrelevant. The three main factorial scores of the PCA from each sample were used to calculate the distance (D) between the two points (populations) under the same treatment P1 = (x_1_, y_1_, z_1_) and P2 = (x_2_, y_2_, z_2_) in 3D space of the PCA (Eq. 1).

1 $$D ( {P_{1} ,\, P_{2} } ) = \sqrt {( {x_{2} - x_{1} } )^{2} + ( {y_{2} - y_{1} } )^{2} + ( {z_{2} - z_{1} } )^{2} }$$

where x, y, and z are the three main factorial scores in the PCA corresponding to the evaluated treatment in Inw and in Cie. Distances were used to evaluate and determine in which salt treatment the greatest differences between the populations were recorded.

## Conclusions

This work shows that cell’s image analysis was efficient at evaluating the salinity-anatomical modification response of *S. europaea* and can be used to identify differences between populations coming from different maternal salinities. By analysing the cell parameters of area and roundness, we can conclude that these parameters are a good indicator of both succulence and plant salinity tolerance. The biochemical analysis proved that anatomical parameters that confer higher salinity tolerance strongly correlate with the cell’s modifications, as confirmed by the Pearson correlation, which highlighted the relationships between anatomical and biochemical parameters. PCA provided evidence that the plants from the anthropogenic saline (Inw) with lower maternal soil salinity (~ 550 mM) habitat are more tolerant to saline stress at laboratory conditions than are those from the natural site with high maternal soil salinity (~ 1000 mM). Our results suggest that the higher salt tolerance of the Inw population may be derived from the maternal salinity being less stressful, and from the better adaptive plasticity of *S. europaea*. Based on our analysis as a whole, it is clear that our applied methods are able to demonstrate that the two *S. europaea* populations from different maternal habitats do indeed have different mechanisms of salt adaptation at a cellular and biochemical level at high salinities, as well as a positive salt-tolerance effect under lower salinities. The gene expression analysis suggested the important role of Na^+^ sequestration into the vacuoles and confirmed that the Inw population can be considered the most salt-tolerant in comparison to Cie. Therefore, these results can be used in the future for the selection of resistant plants. The present correlation results between anatomical and biochemical modifications vs. maternal soil salinity are novel in the study of salt-resistant plants, meaning that researchers can apply this correlation analysis straightforward, for future experiments related to plant salinity-development responses. Although further studies are required, these preliminary results support the idea that maternal effects influence offspring physiology under stress environments. However, future studies are required to consider the ecological context in which plasticity and maternal effects are expressed, such as studies of the patterns of natural populations in term of their environmental heterogeneity.
